# Evaluation of function predictions by PFP, ESG, and PSI-BLAST for moonlighting proteins

**DOI:** 10.1186/1753-6561-6-S7-S5

**Published:** 2012-11-13

**Authors:** Ishita K Khan, Meghana Chitale, Catherine Rayon, Daisuke Kihara

**Affiliations:** 1 Department of Computer Science, Purdue University, 305 N. University Street, West Lafayette, Indiana 47907, USA; 2 Department of Biological Sciences, Purdue University, 915 W. State Street, West Lafayette, Indiana 47907, USA; 3 EA3900-BIOPI Biologie des Plantes et Innovation, Université de Picardie Jules Verne, 33 Rue St Leu, 80039 Amiens, France

## Abstract

**Background:**

Advancements in function prediction algorithms are enabling large scale computational annotation for newly sequenced genomes. With the increase in the number of functionally well characterized proteins it has been observed that there are many proteins involved in more than one function. These proteins characterized as moonlighting proteins show varied functional behavior depending on the cell type, localization in the cell, oligomerization, multiple binding sites, etc. The functional diversity shown by moonlighting proteins may have significant impact on the traditional sequence based function prediction methods. Here we investigate how well diverse functions of moonlighting proteins can be predicted by some existing function prediction methods.

**Results:**

We have analyzed the performances of three major sequence based function prediction methods, PSI-BLAST, the Protein Function Prediction (PFP), and the Extended Similarity Group (ESG) on predicting diverse functions of moonlighting proteins. In predicting discrete functions of a set of 19 experimentally identified moonlighting proteins, PFP showed overall highest recall among the three methods. Although ESG showed the highest precision, its recall was lower than PSI-BLAST. Recall by PSI-BLAST greatly improved when BLOSUM45 was used instead of BLOSUM62.

**Conclusion:**

We have analyzed the performances of PFP, ESG, and PSI-BLAST in predicting the functional diversity of moonlighting proteins. PFP shows overall better performance in predicting diverse moonlighting functions as compared with PSI-BLAST and ESG. Recall by PSI-BLAST greatly improved when BLOSUM45 was used. This analysis indicates that considering weakly similar sequences in prediction enhances the performance of sequence based AFP methods in predicting functional diversity of moonlighting proteins. The current study will also motivate development of novel computational frameworks for automatic identification of such proteins.

## Background

The ever growing genome sequencing data and the overwhelming development of genome sequencing technologies have boosted the development of computational techniques and resources for protein function prediction [1,2]. The traditional sequence based functional annotation is based on the concept of homology [3,4] or motif/domain searches [5-7]. Some recent Automatic Function Prediction (AFP) methods such as PFP [8,9], ESG [10], Gotcha [11], GOFigure [12], and ConFunc [13] use the Gene Ontology (GO) hierarchy. On the other hand, SIFTER [14], FlowerPower [15] and Orthostrapper [16] employ phylogenetic trees to transfer functions to target genes in the evolutionary context. There are other function prediction methods that consider co-expression patterns [17-21], 3D structures of proteins [22-30] as well as protein-protein interaction networks [31-36].

Although existing AFP methods show numerous successful predictions, moonlighting proteins may pose a challenge as they are known to show more than one function that are diverse in nature [37-39]. The varied functional behavior of these proteins can be due to localization within the cell, expression by different cell types, binding of a cofactor, oligomerization, complex formation, or multiple binding sites. Moonlighting proteins have been found to be involved in molecular functions ranging from diseases and disorders [16,40,41] to immune systems [40,41].

In this work, we have analyzed the ability of existing function prediction methods to correctly identify diverse functions of experimentally identified moonlighting proteins [42]. We have collected Gene Ontology (GO) term annotations of these proteins from the UniProt database and manually classified these annotations into two distinct functions. Based on the GO annotations, we have examined the prediction performance of PSI-BLAST and two other major sequence based function prediction methods, the Protein Function Prediction (PFP) and the Extended Similarity Group (ESG) method.

Overall, PFP showed higher average recall than PSI-BLAST and ESG. ESG showed lower recall as compared with PFP and PSI-BLAST, although it has a higher precision. The results suggest that the functional diversity of the moonlighting proteins can be captured if weakly similar sequences are considered among a broad range of similar sequence sets.

## Methods

### Function prediction methods

In this section we briefly describe the three AFP methods we examined, PFP, ESG, and PSI-BLAST. Since PFP [8,9] and ESG [10] have been published in earlier works, please refer to the original works for more details.

### Protein function prediction (PFP) algorithm

The PFP algorithm uses PSI-BLAST to obtain sequence hits for a target sequence and predict GO function annotations. PFP computes the score to GO term *f_a _*as follows:

(1) $$s\left(fa\right)=\overset{N}{\underset{i=1}{\sum }}\overset{Nfunc\left(i\right)}{\underset{j=1}{\sum }}\left(\left(-\text{log}\left(E\text{\_}value\left(i\right)\right)+b\right)P\left(fa|fj\right)\right),$$

where *N *is the number of sequence hits considered in the PSI-BLAST hits up to E-value of 100, *Nfunc(i) *is the number of GO annotations for the sequence hit *i, E_value(i) *is the PSI-BLAST E_value for the sequence hit *i, f_j _*is the *j*-th annotation of the sequence hit *i*, and constant *b *takes value *2 (= log_10_100) *to keep the score positive as retrieved sequences up to E_value of 100 are used (-log(E_value(i)) + b = -*log*_10_(100) + 2 = 0, when E_value = 100). The conditional probabilities *P(f_a_|f_j_) *is to consider co-occurrence of GO terms in single sequence annotation, which is computed as the ratio of number of proteins co-annotated with GO terms *f_a _*and *f_j _*as compared with genes annotated with the term *f_j_*. To take into account the hierarchical structure of the GO, PFP transfers the raw score to the parental terms by computing the proportion of proteins annotated with *f_a _*relative to all proteins that belong to the parental GO term in the database. The score of a GO term computed as the sum of the directly computed score by Eqn. 1 and the ones from the parental propagation is called the raw score.

### Extended Similarity Group (ESG) algorithm

ESG recursively performs PSI-BLAST searches from sequence hits obtained in the initial search from the target sequence, thereby performing multi-level exploration of the sequence similarity space around the target protein. Each sequence hit in a search is assigned a weight that is computed as the proportion of the -log(E_value) of the sequence relative to the sum of -log(E_value) from all the sequence hits considered in the search of the same level. This weight is assigned for GO terms annotating the sequence hit. The weights for GO terms found in the second level search are computed in the same fashion. Ultimately the score for a GO term is computed as the total weight from the two levels of the searches. The score for each GO term ranges from 0 to 1.0.

### PSI-BLAST

PSI-BLAST search is performed with a default setting with maximum of three iterations. Then the top hits with an E_value score better than 0.01 that have annotations is used for transferring annotation to the query sequence. The BLAST predictions were ranked according to -log(E_value)+2 for each of the prediction. In addition to the default BLOSUM62, which is the default amino acid similarity matrix, we also tested PSI-BLAST performance using BLOSUM45 and BLOSUM30.

## Results

We analyzed the performances of PFP, ESG, and PSI-BLAST in predicting the functional diversity of 19 moonlighting proteins. The 19 moonlighting proteins were taken from a review article [42]. These proteins have two diverse and distinct functions. According to the verbal description of the two diverse functions of the proteins, we classified GO terms of these proteins in UniProt into four classes: Terms that belong to the major moonlighting function of the protein (Function 1); those which belong to the second moonlighting function (Function 2); terms which belong to both functions; and terms that do not belong to either of the functions. The list of the moonlighting proteins and their classified GO terms are made available at http://kiharalab.org/MoonlightingProtein_Dataset1/.

The raw score of PFP predictions has a large range of values. Up to 1000 GO term predictions were sorted by their raw score and plotted at an interval of 10. ESG predictions have a score range of 0 to 1.0, and 100 cutoffs are used within this range. PSI-BLAST predictions are ranked by -log(E_value)+2, and 100 score cutoffs are used from 4 (E_value of 0.01) to 45 (E_value of 10^-43^). To compare the prediction performances of the methods, we computed precision and recall. Precision is defined as TP/(TP+FP) and recall is defined as TP/(TP+FN), where TP and FP denote true and false positive, respectively, and FN denote false negative. All predictions by the three methods are propagated to the root of the GO hierarchy, so are the true annotations for the proteins.

### Average Precision-Recall performance of PFP, ESG, and PSI-BLAST

**Figure 1:**
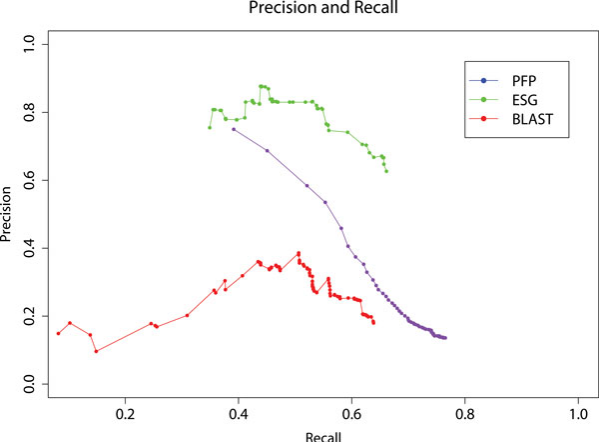
**Precision-Recall of PFP, ESG, and PSI- BLAST**.

**Figure 2:**
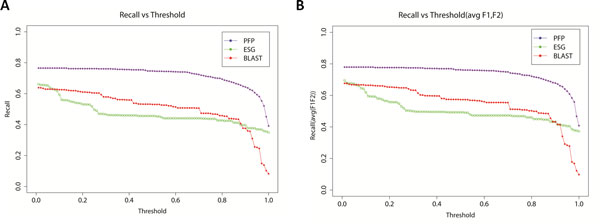
**Recall of PFP, ESG and PSI-BLAST at each threshold**. **A**, Recall where all the GO annotations for proteins are considered. **B**, Recall where only the GO annotations labeled as Function 1 or Function 2 for proteins are considered.

In Figure 1, the average precision and recall of PFP, ESG, and PSI-BLAST for all the GO terms of the 19 moonlighting proteins are shown. It is shown that ESG perform significantly better than the other two methods in the recall range of 0.4 - 0.7. ESG has better precision than BLAST within recall range of 0.37 - 0.66. PFP predictions ranked with raw score (Eq. 1 in Methods) reaches the highest recall. In Figure 2 we show the performance of the methods in terms of recall values at 100 cutoff scores (with all the GO annotations of the proteins considered). It is apparent from this plot that PFP showed higher recall than PSI-BLAST, and ESG. ESG has lowest recall within the cutoff range of 0.09-0.88.

In Figure 2B, the performance was evaluated where only the GO annotations for the two moonlighting functions (Function 1 and Function 2) are taken into account as the target annotations. The prediction performance for the moonlighting functions is essentially the same as those measured for the all GO term annotations (Figure 2A).

### Recall at individual proteins

**Figure 3:**
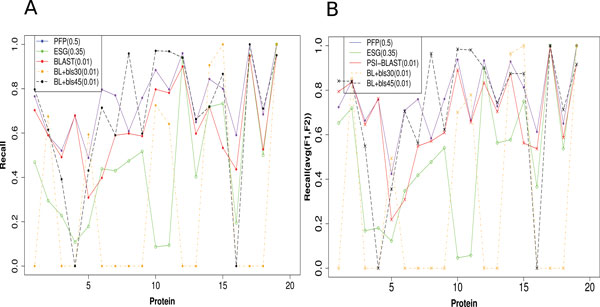
**Recall of PFP, ESG, PSI-BLAST, PSI-BLAST with BLOSUM62 (default), BLOSUM30, and BLOSUM45 scoring matrix for each protein**. Score thresholds used for the methods are PFP: 0.5, ESG: 0.35 and PSI-BLAST: 0.01 **A**, Recall where all the GO annotations for proteins are considered. **B**, Recall where only the GO annotations labeled as Function 1 or Function 2 for proteins are considered.

Next In Figure 3, we plotted the recall for the three methods for each of the 19 moonlighting proteins separately. The cutoff of the prediction scores used are 0.5 for PFP, 0.35 for ESG, and E_value 0.01 for PSI-BLAST. The PFP cutoff of 0.5 will yield the maximum of 500 GO term predictions. The score cutoff value of 0.35 for ESG is an optimal cutoff score established in the previous work [10]. E_value 0.01 for PSI-BLAST is a standard cutoff used in general for homology search. In addition to default PSI-BLAST setting with BLOSUM62, we have also added the predictions of two more versions of PSI-BLAST, with BLOSUM45 and BLOSUM30 scoring matrices (BL+bls45 and BL+30 in Figure 3, respectively) to consider more divergent sequences in the homology search.

When all the GO terms are considered (Figure 3A), PFP showed higher recall than PSI-BLAST for almost all the cases (except for proteins 2 and 4, which are ties). ESG has similar recall of predictions as PSI-BLAST for proteins 14 and 17, slightly higher recall for proteins 6, 12 and 15 than PSI-BLAST (BLOSUM62), and a lower recall than PFP and PSI-BLAST for the rest of the proteins. Recall by PSI-BLAST improved when BLOSUM45 was used. In the head-to-head comparison against PFP, PSI-BLAST with BLOSUM45 showed a higher recall than PFP for eight proteins while PFP had a higher recall in ten cases (there was a tie). PSI-BLAST with BLOSUM30 failed to predict any GO terms above E_value of 0.01 for twelve proteins (Figure 3A). Overall, PFP and PSI-BLAST with BLOSUM45 showed higher recall than the rest of the methods. We see a similar performance pattern for the five methods when we consider only the GO terms belonging to moonlighting function 1 and function 2 of the proteins (Figure 3B). Again PSI-BLAST with BLOSUM45 showed comparable performance to PFP. PSI-BLAST with BLOSUM45 had a higher recall than PFP in seven cases while PFP was higher in ten cases (again there was a tie).

These results indicate that the PFP can find moonlighting GO terms that are missed by regular PSI-BLAST searches for quite a lot of cases. The strength of PFP is its coverage of a large number of sequences, by including weakly similar sequences into consideration for annotation transfer. On the other hand, ESG puts more weight on the consensus sequences that have strong similarity with the query protein among all the sequences that it encounters along multiple iterations. Thus, although ESG provides a higher precision on the predictions among all three methods (Figure 1), it fails to detect the functional variations in a number of cases. These results suggest that the functional diversity of the moonlighting proteins could be captured by taking weakly similar sequences into consideration among a broad range of similar sequences.

## Conclusion

The identification of moonlighting functions of a protein is important for automatic function predictions. We have analyzed the performances of PFP, ESG, and PSI-BLAST in predicting the functional diversity of moonlighting proteins. PFP shows overall better performance in predicting diverse moonlighting functions as compared with PSI-BLAST and ESG. Recall by PSI-BLAST greatly improved when BLOSUM45 was used instead of BLOSUM62. This analysis indicates that considering weakly similar sequences in prediction enhances the performance of sequence based AFP methods in predicting functional diversity of moonlighting proteins.

## Competing interests

The authors declare that they have no competing interests.

## Authors' contributions

IKK did the experiment and drafted the manuscript. MC has developed ESG and helped in doing the experiment. CR has participated in classifying GO terms of the moonlighting proteins. DK conceived the study and participated in its design and coordination, as well as drafting and finalizing the manuscript. All authors read and approved the final manuscript.

## References

[B1] Hawkins T Kihara D Function prediction of uncharacterized proteins Journal of bioinformatics and computational biology 2007 5 1 30 10.1142/S0219720007002503 17477489

[B2] Hawkins T Chitale M Kihara D New paradigm in protein function prediction for large scale omics analysis Mol BioSyst 2008 4 223 231 10.1039/b718229e 18437265

[B3] Altschul SF Gish W Miller W Myers EW Lipman DJ Basic local alignment search tool Journal of molecular biology 1990 215 403 410 2231712 10.1016/S0022-2836(05)80360-2

[B4] Pearson WR Rapid and sensitive sequence comparison with FASTP and FASTA Methods in enzymology 1990 183 63 98 2156132 10.1016/0076-6879(90)83007-v

[B5] Bru C Courcelle E Carrere S Beausse Y Dalmar S Kahn D The ProDom database of protein domain families: more emphasis on 3D Nucleic acids research 2005 33 D212 D215 15608179 10.1093/nar/gki034 PMC539988

[B6] Finn RD Mistry J Schuster-Bockler B Griffiths-Jones S Hollich V Lassmann T Pfam: clans, web tools and services Nucleic acids research 2006 34 D247 D251 10.1093/nar/gkj149 16381856 PMC1347511

[B7] Hunter S Apweiler R Attwood TK Bairoch A Bateman A Binns D InterPro: the integrative protein signature database Nucleic acids research 2009 37 D211 D215 10.1093/nar/gkn785 18940856 PMC2686546

[B8] Hawkins T Luban S Kihara D Enhanced automated function prediction using distantly related sequences and contextual association by PFP Protein Science 2006 15 1550 1556 10.1110/ps.062153506 16672240 PMC2242549

[B9] Hawkins T Chitale M Luban S Kihara D PFP: automated prediction of gene ontology functional annotations with confidence scores using protein sequence data Proteins: Structure, Function, and Bioinformatics 2009 74 566 582 10.1002/prot.22172 18655063

[B10] Chitale M Hawkins T Park C Kihara D ESG: extended similarity group method for automated protein function prediction Bioinformatics 2009 25 1739 1745 10.1093/bioinformatics/btp309 19435743 PMC2705228

[B11] Martin D Berriman M Barton G GOtcha: a new method for prediction of protein function assessed by the annotation of seven genomes BMC Bioinformatics 2004 5 178 194 10.1186/1471-2105-5-178 15550167 PMC535938

[B12] Khan S Situ G Decker K Schmidt CJ GoFigure: automated Gene Ontology annotation Bioinformatics 2003 19 2484 2485 10.1093/bioinformatics/btg338 14668239

[B13] Wass MN Sternberg MJ ConFunc--functional annotation in the twilight zone Bioinformatics 2008 24 798 806 10.1093/bioinformatics/btn037 18263643

[B14] Engelhardt BE Jordan MI Muratore KE Brenner SE Protein molecular function prediction by Bayesian phylogenomics PLoS Comput Biol 2005 1 e45 10.1371/journal.pcbi.0010045 16217548 PMC1246806

[B15] Krishnamurthy N Brown D Sj+¦lander K FlowerPower: clustering proteins into domain architecture classes for phylogenomic inference of protein function BMC Evolutionary Biology 2007 7 S12 17288570 10.1186/1471-2148-7-S1-S12 PMC1796606

[B16] Storm CEV Sonnhammer ELL Automated ortholog inference from phylogenetic trees and calculation of orthology reliability Bioinformatics 2002 18 92 10.1093/bioinformatics/18.1.92 11836216

[B17] Brown MPS Grundy WN Lin D Cristianini N Sugnet CW Furey TS Knowledge-based analysis of microarray gene expression data by using support vector machines Proceedings of the National Academy of Sciences 2000 97 262 10.1073/pnas.97.1.262 PMC26651 10618406

[B18] Eisen MB Spellman PT Brown PO Botstein D Cluster analysis and display of genome-wide expression patterns Proceedings of the National Academy of Sciences 1998 95 14863 10.1073/pnas.95.25.14863 PMC24541 9843981

[B19] Gao L Li X Guo Z Zhu M Li Y Rao S Widely predicting specific protein functions based on protein-protein interaction data and gene expression profile Sci China C Life Sci 2007 50 125 134 10.1007/s11427-007-0009-1 17393093

[B20] Khatri P Dr-âghici S Ontological analysis of gene expression data: current tools, limitations, and open problems Bioinformatics 2005 21 3587 3595 10.1093/bioinformatics/bti565 15994189 PMC2435250

[B21] van Noort V Snel B Huynen MA Predicting gene function by conserved co-expression TRENDS in Genetics 2003 19 238 242 10.1016/S0168-9525(03)00056-8 12711213

[B22] Gherardini PF Helmer-Citterich M Structure-based function prediction: approaches and applications Briefings in functional genomics & proteomics 2008 7 291 302 10.1093/bfgp/eln030 18599513

[B23] Marti-Renom M Rossi A Al-Shahrour F Davis F Pieper U Dopazo J The AnnoLite and AnnoLyze programs for comparative annotation of protein structures BMC Bioinformatics 2007 8 S4 17570147 10.1186/1471-2105-8-S4-S4 PMC1892083

[B24] Martin ACR Orengo CA Hutchinson EG Jones S Karmirantzou M Laskowski RA Protein folds and functions Structure 1998 6 875 884 10.1016/S0969-2126(98)00089-6 9687369

[B25] Pal D Eisenberg D Inference of protein function from protein structure Structure 2005 13 121 130 10.1016/j.str.2004.10.015 15642267

[B26] Ponomarenko JV Bourne PE Shindyalov IN Assigning new GO annotations to protein data bank sequences by combining structure and sequence homology Proteins: Structure, Function, and Bioinformatics 2005 58 855 865 10.1002/prot.20355 15645518

[B27] Thornton JM Todd AE Milburn D Borkakoti N Orengo CA From structure to function: approaches and limitationsnature structural biology 2000 7 991 994 10.1038/80784 11104008

[B28] Chikhi R Sael L Kihara D Real-time ligand binding pocket database search using local surface descriptors Proteins: Structure, Function, and Bioinformatics 2010 78 2007 2028 10.1002/prot.22715 PMC3009464 20455259

[B29] Sael L Kihara D Binding ligand prediction for proteins using partial matching of local surface patches International Journal of Molecular Sciences 2010 11 5009 5026 10.3390/ijms11125009 21614188 PMC3100846

[B30] Sael L Chitale M Kihara D Structure- and sequence-based function prediction for non-homologous proteins. Journal of Structural and Functional Genomics. Journal of Structural and Functional Genomics 2012 Ref Type: In Press 10.1007/s10969-012-9126-6 PMC3375349 22270458

[B31] Brun C Chevenet F Martin D Wojcik J Guenoche A Jacq B Functional classification of proteins for the prediction of cellular function from a protein-protein interaction network Genome Biol 2003 5 R6.1 R6.13 14709178 10.1186/gb-2003-5-1-r6 PMC395738

[B32] Chua HN Sung WK Wong L Exploiting indirect neighbours and topological weight to predict protein function from protein-protein interactions Bioinformatics 2006 22 1623 1630 10.1093/bioinformatics/btl145 16632496

[B33] Letovsky S Kasif S Predicting protein function from protein/protein interaction data: a probabilistic approach Bioinformatics 2003 19 Suppl 1 i197 i204 10.1093/bioinformatics/btg1026 12855458

[B34] Nariai N Kolaczyk ED Kasif S Probabilistic protein function prediction from heterogeneous genome-wide data PLoS One 2007 2 e337.1 e337.7 17396164 10.1371/journal.pone.0000337 PMC1828618

[B35] Sharan R Ulitsky I Shamir R Network-based prediction of protein function Mol Syst Biol 2007 3 88 100 17353930 10.1038/msb4100129 PMC1847944

[B36] Deng M Tu Z Sun F Chen T Mapping gene ontology to proteins based on protein-protein interaction data Bioinformatics 2004 20 895 902 10.1093/bioinformatics/btg500 14751964

[B37] Jeffery CJ Moonlighting Proteins Trends in Biochemical Sciences 1999 24 8 11 10.1016/S0968-0004(98)01335-8 10087914

[B38] Jeffery CJ Moonlighting Proteins: old proteins learning new tricks TRENDS in Genetics 2003 19 415 417 10.1016/S0168-9525(03)00167-7 12902157

[B39] Gancedo C Flores CL Moonlighting proteins in yeasts Microbiology and Molecular Biology Reviews 2008 72 197 210 10.1128/MMBR.00036-07 18322039 PMC2268286

[B40] Jeffery CJ Proteins with neomorphic moonlighting functions in disease IUBMB Life 2011 63 489 494 10.1002/iub.504 21698752

[B41] Ovadi J Moonlighting Proteins in Neurological Disorders IUBMB Life 2011 63 453 456 10.1002/iub.491 21698748

[B42] Huberts DHEW Klei IJvd Moonlighting proteins: an intriguing mode of multitasking Biochim Biophys Acta 2010 1803 520 525 10.1016/j.bbamcr.2010.01.022 20144902

